# Personalized nutrition: the end of the one-diet-fits-all era

**DOI:** 10.3389/fnut.2024.1370595

**Published:** 2024-05-24

**Authors:** Sonia Roman, Liliana Campos-Medina, Leonardo Leal-Mercado

**Affiliations:** ^1^Department of Genomic Medicine in Hepatology, Civil Hospital of Guadalajara, Fray Antonio Alcalde, Guadalajara, Jalisco, Mexico; ^2^Health Sciences Center, University of Guadalajara, Guadalajara, Jalisco, Mexico; ^3^Doctoral Program in Molecular Biology in Medicine, Health Sciences Center, University of Guadalajara, Guadalajara, Jalisco, Mexico

**Keywords:** genes, polymorphisms, ancestry, Genomex diet, hepatopathogenic diet, food culture, Latin America, Mexico

## Abstract

Personalized Nutrition emerged as a new trend for providing nutritional and food advice based on the individual’s genetic composition, a field driven by the advancements in the multi-omic sciences throughout the last century. It intends not only to tailor the recommended daily allowances of nutrients and functional foods that a person may need but also to maintain the principles of sustainability and eco-friendliness. This principle implies the implementation of strategies within the healthcare system to advocate for the ending of the one-diet-fits-all paradigm by considering a personalized diet as an ally to prevent diet-related chronic diseases. In this Perspective, we highlight the potential benefits of such a paradigm within the region of Latin America, particularly Mexico, where the genetic admixture of the population, food biodiversity, and food culture provide unique opportunities to establish personalized nutrigenetic strategies. These strategies could play a crucial role in preventing chronic diseases and addressing the challenges confronted in the region.

## 1 Introduction

The field of Genomic Nutrition, also known as Nutritional Genomics, is rapidly advancing with the integration of multi-omic analyses in nutritional science (1, 2). This discipline mainly encompasses two subfields, Nutrigenomics and Nutrigenetics, which explore the bidirectional interactions between genes, diet (nutrients), and health outcomes (3, 4). Nutrigenetics focuses on how a specific polymorphism (allele/genotype) or genetic profile affects the body’s metabolic responsiveness to foods (nutrients) (5). Nutrigenomics studies the impact of nutrients and bioactive food compounds on gene expression, specifically in transcriptomics, proteomics, and metabolomics, regardless of the inherited genotype (6). In addition, the emerging fields of Nutri-epigenetics and Nutri-metagenomics widen our understanding of gene expression modulation at the chromatin level (7, 8) and the signaling between the gut microbiota, considered our “second genome,” and the host’s organs/tissues (9, 10), respectively. Other interacting environmental factors to consider are physical activity, psychosocial context (stress, emotions), and contaminants (11). Together, they provide the scientific basis for designing more effective dietary interventions that consider genetic diversity, gut microbiota, and lifestyle to design personalized nutrition strategies that align with an individual’s unique needs.

Research in Nutritional Genomics and its practical application in personalized nutrition have incited two interconnected aspects. On the one hand, much enthusiasm has risen due to the potential to predict nutritional recommendations based on individual genetic profiles, leading to improved health outcomes (12). In addition, understanding the genomic landscape contributing to the risk of chronic diseases in a specific population has advantages. It allows for proactive measures to prevent these diseases rather than relying solely on reactive healthcare (13, 14). On the other hand, the inception of personalized nutrition based on multi-omic data has sparked a significant debate among health experts regarding the interpretation of this data and ethical and privacy concerns surrounding it (15). These controversies have led to the establishment of standardized definitions, regulations, and ethical delivery of nutritional care (16). Although crucial for ensuring professional clinical practices, these aspects go beyond the scope of this Perspective. Nonetheless, it is undeniable that personalized nutrition, indicating the right food for all, is an ongoing trend in our society (17, 18) that will inherently lead to the end of the universal approach of the “one-diet-fits-all era”.

Herein, we examine our rationale and the implications of implementing personalized nutrition in Latin America, particularly Mexico. The population’s varying genetic backgrounds, food diversity, and cultural traditions present a potential for tailoring regionalized nutritional genomic strategies to move away from a “one-diet-fits-all” to fight chronic illnesses.

## 2 Evolution of the human diet and mismatch between genes and nutrients

Gene-nutrient interactions are highly dynamic. Firstly, there have been significant evolutionary changes in how humans obtain nutrients from the environment and how these needs are regulated by genes (19). Dietary patterns have substantially changed across different regions throughout history (20). Currently, numerous societies are amid a shift from pre- and post-globalization movements. This transition has resulted in detrimental effects on food quality and quantity, as well as an increase in chronic diseases due to greater food processing and accessibility (21, 22). Likewise, humans have experienced different stages of evolutionary adaptation through exposure to various environments and selective pressures. The genome of modern humans has been shaped by the availability of nutrients in different environments, leading to multiple locally positively selected gene variations (23). While advantageous in one setting, these adaptations can become problematic when faced with a changing nutritional landscape, such as in the current epidemiological transition (24–26). The significant shifts in lifestyle factors, such as reduced physical activity, increased stress levels, and exposure to environmental pollutants, are causing an evolutionary mismatch that contributes to the development of chronic illnesses (26).

From a nutritional standpoint, some societies maintain a historically traditional diet, while others have adopted either an imported traditional diet or a more modern diet (27). Unfortunately, this does not suggest that our overall health surpasses that of our ancestors or previous generations. The prevalence of obesity-related chronic diseases has increased globally, affecting individuals of all economic backgrounds. This trend is particularly evident among children, adolescents, and adults residing in urban areas where ultra-processed foods have surged alongside the acculturation process (28, 29).

In contrast, sustaining or reintroducing indigenous food knowledge and protecting traditional cultural food practices worldwide positively impacts social-cultural well-being and substantially reduces the likelihood of developing chronic diseases (30). The composition of traditional diets, which refers to the predominant diet consumed for many generations and comprises a higher share of natural staple foods, reflects earlier phases of food evolution before industrialization (31). Hence, personalized nutrition should focus on reintroducing the main staple foods that have influenced human DNA in the past. However, these foods are not universal and will vary significantly according to geography, the population’s ethnicity, and cultural practices.

## 3 Personalized nutrition to prevent diet-related chronic diseases

In the early days before the genomics era, Dr. Richard O. Brennan introduced the concept of nutrigenetics in 1977. He used this term to describe the notion that diet could alleviate hypoglycemia, which is linked to genetics (32). In addition, newborns that inherit single-gene inborn metabolism errors and diseases require personalized nutritional support to address these diseases at early stages (33). However, the boom of personalized nutrition, as we recognize it today, occurred during the post-genomic era (6). The Human Genome Project gained significant attention because it became clear that most common chronic disease phenotypes derive from the complex interplay between multiple genetic variations, (single nucleotide polymorphisms (SNPs), number copy variations and insertion-deletion polymorphisms) and environmental factors such as diet, containing nutrients and bioactive compounds (34, 35). The Human Microbiome Project was subsequently established to disseminate knowledge, resources, and discoveries that connect human-microbiome interactions and health-related outcomes (36).

Personalized nutrition or precision nutrition multi-omic technologies have ultimately revolutionized healthcare strategies by recognizing the unique nature of individuals and the need for tailored dietary recommendations. Nevertheless, it is crucial not to disregard a fundamental principle. Personalized nutrition should consider the occurrence of the diseases it aims to prevent, which are, in turn, shaped by genetic and cultural factors specific to the target population (37, 38). Hence, it is not only the inheritance of “risk alleles” that is important, but also the traditions and history (food culture) that shaped that genome, so diets should not be recommended indiscriminately. Developing personalized nutrition plans is and will be a complex task, but it is feasible due to the growing scientific research, societal acceptance, political backing, and health and economic policies (39, 40).

## 4 The one-diet-fits-all saga

Good nutrition is fundamental in sustaining a healthy lifestyle across all stages of human development (41). Preventing chronic diseases is crucial because life expectancy has increased, and living disease-free enhances the quality of the aging process (42–44). In the past, mothers had an important role in feeding, and nourishment was delivered by moms’ dietary approaches, which influenced the food environment and shaped our eating patterns within the family (45, 46). Family recipes, influenced by the geographic availability of food resources, transmit wisdom to the younger generations by guiding the consumption of essential foods and nutrients and cooking practices (food culture).

Maintaining good habits throughout life can contribute to one’s overall health. It is worth noting that prior to industrialization, most individuals’ primary cause of death was infectious diseases rather than nutrition-related chronic diseases (47). While there may be some overlap between undernutrition and overnutrition, human dietary habits have culturally shifted regarding the types, locations, and quantities of food we consume, which differs from the biological needs encoded in our genes. Following “mom’s advice” has become increasingly difficult due to shifts in the food system, insufficient personalization of dietary recommendations, and the promotion of non-regional cuisines.

The convergence of multiple tendencies may have endorsed the one-diet-fits-all regimen. First, globalization of the food supply facilitated the importation of highly or ultra-processed products into underdeveloped countries or their targeted marketing to underprivileged sectors, chiefly because of their affordability and widespread availability (48). Globalization has increased the likelihood of societies to prefer “globalized” food that may differ in quality from locally produced foods. Secondly, several health organizations in the United States reached a consensus on the recommended guidelines for essential nutrients such as carbohydrates, proteins, fats, vitamins, antioxidants, minerals, and fiber to prevent atherosclerosis, cancer, diabetes, and obesity (49). As previously stated, the need to reverse the growing prevalence of chronic diseases led to the unification of standard dietary guidelines for the clinical management of chronic-diseased patients without considering genetic or cultural factors, endorsing a one-diet-fits-all approach (50). Thirdly, the recommendation to adopt traditional diets such as the “Mediterranean diet,” “Japanese diet,” or “Nordic diet” (51–53) to lower the onset of chronic disease outside their region of origin overlooks the equally beneficial nutritional and economic advantages of local diets (54). All things considered, one type of local diet is not healthier or better than any other, and trying to adapt specific diets to resemble, for example, the Mediterranean diet as a universal diet to treat chronic disease (55, 56) is against the required food system, food culture, or a personalized nutrition approach.

Furthermore, when incorporated into national clinical practice guidelines, these exotic dietary programs may not be practical for most people or suitable for their genetic composition (57). Furthermore, parallel to the criticism against the one-diet-fits-all trend, there is a need for tailored “normal” cut-off values of health indicators regarding body mass index, glucose levels, liver function tests, or fat percentage to account for the variability in human body measurements. Thus, in a broader sense, the personalized nutrition movement is an opportunity to provide real-life population-based or regionalized recommendations for societies seeking to prevent chronic diseases based on their characteristics.

## 5 Shaping the basis of personalized nutrition in Mexico

Research has revealed that human populations possess genetic variations that enable them to withstand better extreme climates, high altitudes, infectious diseases, or varying levels of nutrient availability. As mentioned before, these adaptations are specific to local environments (23). However, there is limited knowledge regarding the nutritional adaptations of Latin American populations, including Mexico, and the mechanisms behind their development. Thus, our research has focused on developing a comprehensive biocultural model of genome-based nutrition integrating the individual’s or population’s genetic background with their food culture to create personalized nutritional recommendations or interventions (58, 59).

The genetic history of Mexico traces back to the arrival of the Amerindians or First Nations People, who exploited the rich ambient biodiversity of the region. The early events after the Spanish conquest involved the introduction of European and African genes, which now form the mixed genetic pool of the present population. Additionally, there was an exchange of Old and New World foods, leading to the development of a distinct regional food culture heritage and subsequent food preferences. However, before European colonialism, the Amerindian tribes inhabited distinct ecological regions. Aridoamerica, located in the northern part of Mexico, was the land of nomadic hunter-gatherers, and Mesoamerica, spanning from central Mexico to part of Nicaragua, was home to sedentary/agricultural ethnic groups (60). Recent studies reveal that the Mexican Indigenous people exhibit signatures of local adaptations resulting from their specific dietary and cultural practices. These adaptations have influenced their biological networks, substrate metabolism, and disease susceptibility (61, 62).

**Figure 1:**
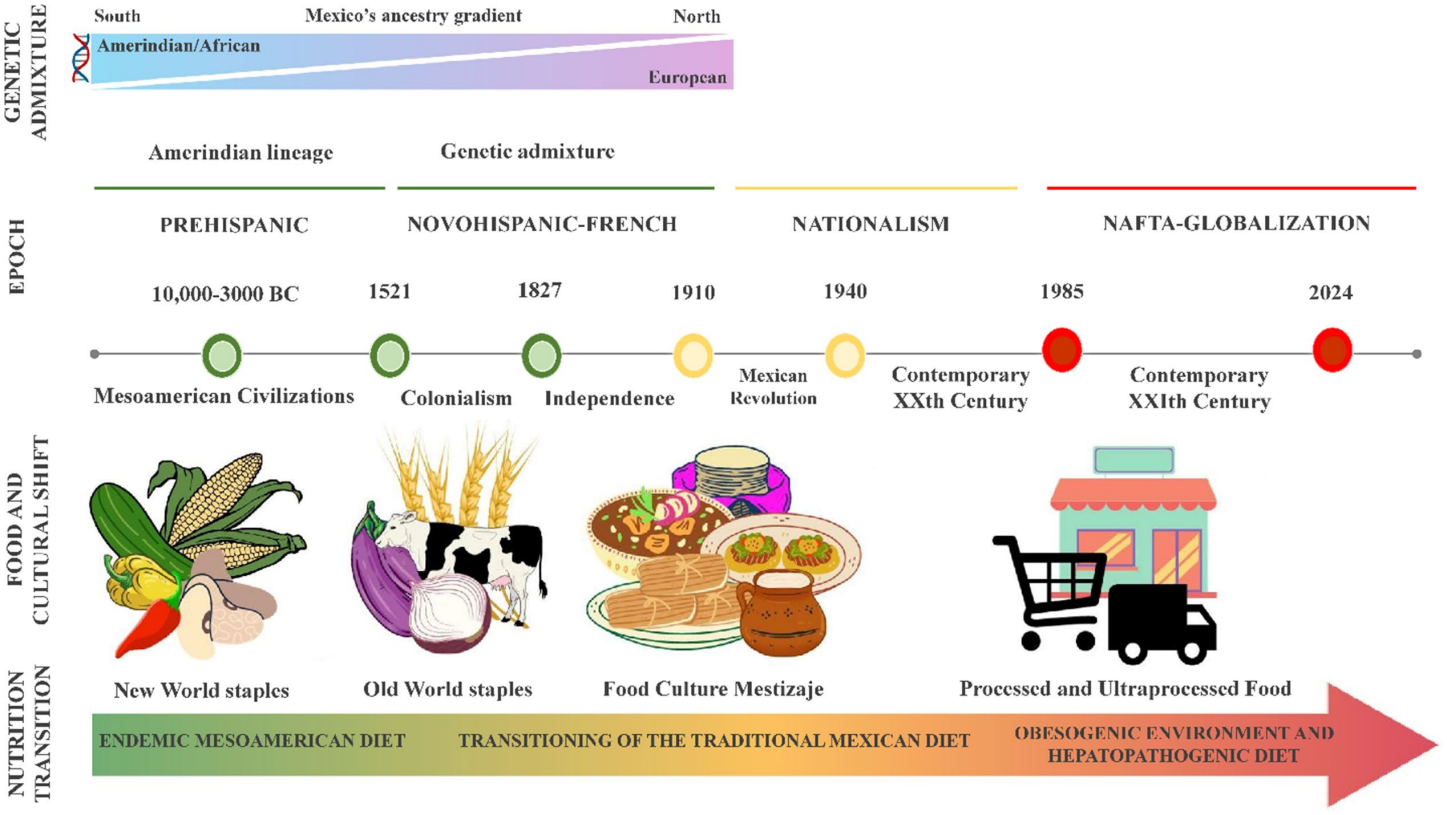
Genetic and alimentary evolution of the Mexican population. Mexico’s population genetics and food culture have undergone significant shifts with each economic/historical period (herein marked illustratively), shaping dietary preferences and influencing susceptibility to infectious and chronic diseases. This timeline highlights the shift from harmony with the environment to a growing mismatch between genes and the modern diet. The endemic Mesoamerican diet, rich in plant-based staples like maize, beans, and chili, was historically linked to lower metabolic risk factors. However, this has been replaced by a highly palatable, energy-dense diet dominated by processed and ultra-processed foods, poor nutritional quality, and promoting metabolic diseases. In this obesogenic environment, Mexico faces an obesity epidemic among its young and adult population, fueled by dietary patterns that result in metabolic disturbances like dyslipidemia, insulin resistance, diabetes, and metabolic-associated steatotic liver disease (MASLD). NAFTA, North American Free Trade.

Figure 1 illustrates the transformative changes in Mexico’s genetics and food culture throughout the last five centuries. These transitions have played a crucial role in shaping dietary preferences and impacting the population’s vulnerability to infectious and chronic diseases. The period of Spanish rule led to the admixture of the European, Amerindian, and African lineages with a gradient shift in the ancestral components from north to south (63). This feature translates into the fact that North Mexicans with mixed ancestry could benefit from tailored recommendations based on their higher European heritage, contrasting with individuals from the South with Amerindian ancestry, as discussed later (64–66).

The Mesoamerican food system constitutes the foundation of the traditional Mexican diet, primarily consisting of plant-based foods such as maize, beans, chili, squash, tomato, chia, pumpkin seeds, amaranth, prickly pears, and cacao. Research has shown that this diet is associated with a reduced risk of metabolic disorders and is nutritionally well-balanced and culturally acceptable (67). Nevertheless, the Mexican population is immersed in an obesogenic environment, consuming a hepatopathogenic diet that causes dyslipidemias, insulin resistance, steatosis, and metabolic-associated steatotic liver disease (MASLD), formerly known as non-alcoholic fatty liver disease (NAFLD) (65, 68–70). As shown in Figure 1, the transition from a state of evolutionary concordance to a mismatch between the nature of our ancestral genes and the environment is part of the current health problem. Based on this evidence, a comprehensive approach was developed to address the prevalent health issues within the Mexican population.

## 6 The Genomex diet: benefits and challenges

The evidence mentioned above is substantial enough to establish a framework for implementing a public health policy that considers the balance between ancestral genes and our food options. Paradoxically, the battle against the prevailing Westernized diet and its detrimental effects is fought by advocating for the “gold standard” Mediterranean diet as if it were *the silver bullet*. Almost all major clinical practice guidelines in Mexico, endorsed by the medical associations, recommend adopting the Mediterranean diet to prevent chronic diseases and overlook the fundamental national dietary recommendations (71). With this attitude, we fail to seize the valuable opportunity to offer nutritious, environmentally friendly, and sustainable food choices to a population immersed in an obesity epidemic and with financial constraints that prevent them from purchasing expensive non-local ingredients regularly.

Even though the traditional Mediterranean and Mexican diets are culturally inherited eating patterns that consist of distinct recipes made using locally sourced ingredients and are highly valued in each society, they are not head-to-head comparable (72). Established initially on earlier agricultural and rural models, the Mediterranean diet reflects the illustrious history of culinary and cultural exchanges that have occurred in the countries surrounding the Mediterranean Basin for millennia (73). This diet contains wheat-based (bread, pasta, or couscous) foods, a wide range of plant-based foods, and virgin olive oil as the primary source of fat. It also includes a moderate intake of red wine, seafood, fermented dairy products, poultry, and eggs and a low consumption of red and processed meat and sweets (74, 75). On the contrary, the traditional Mexican dietary regimen predominantly comprises Mesoamerican staples cultivated in this area. These include maize and its by-products, such as “tortillas,” legumes (beans), high amounts of vegetables (e.g., dark-green leafy vegetables named “quelites,” squash, tomato, chile, and prickly pears), and plant-based fats obtained from avocado, chia, pumpkin seeds, and amaranth. Complementary foods include fruits, beverages (e.g., “cacao,” “pulque,” and “tesgüino” fermented beverages), fish and seafood, small wild animal meat, herbs, and condiments (76). Therefore, it is worth noting that Mexico can potentially promote the components of the Mesoamerican diet and the traditional postcolonial dishes as healthy dietary options (67), whereas the Mediterranean diet is not entirely feasible.

Furthermore, the traditional Mexican diet received the prestigious recognition of the Intangible Cultural Heritage in 2010 (77). This distinction is valuable for educating the public about this cultural legacy’s significance rather than merely being displayed on a wall. The healthcare community frequently stigmatizes the typical Mexican diet as being high in fat and contributing to weight gain. However, the root issue lies in the overconsumption of modern, non-native, calorie-dense processed foods and the lack of nutrition education.

Genomic analyses conducted on Mexicans have provided valuable insights into the influence of ancestry on the population’s health (62, 66, 78) and the potential consequences of not implementing preventive measures to mitigate disease risks. In light of the significant rise in unhealthy eating habits in Mexico and the growing prevalence of obesity and associated co-morbidities, we set forth to develop a strategy to prevent and address the metabolic alterations driven by the gen-environment mismatch.

To this end, the next step was to create a genome-based nutrition program aligning with the Mexican population’s genetic background and food culture, denoted as the Genomex diet. Despite its trendy name, the diet is not meant to be a fad diet because it adheres to the principles of a correct diet, and it seeks to promote the consumption of nutritious staples that are culturally accepted while considering regional genetic and culinary variations. The Genomex diet combines the nutritional advantages of traditional local dishes, which contain Mesoamerican staple foods rich in nutrients and bioactive components that are prepared in a healthy manner and align with the Mexican population’s functional nutrigenetic and nutrigenomic characteristics (58).

Table 1 provides an overview of the genetic polymorphisms and their evolutionary mismatch, which the Genomex diet intends to counteract, highlighting the adaptive alleles that become “risk alleles” due to unhealthy eating habits. For example, the high frequency of the *MTHFR* 677 T allele among the Amerindian population is consistent with their historical dietary habits of consuming folate-rich leafy greens (64). In addition, admixed subpopulations with a higher percentage of Amerindian ancestry are more likely to carry the 677 T allele than those with European ancestry, predominantly carriers of the 677C allele. Furthermore, a correlation has been observed between the 677 T risk allele and the presence of liver steatosis and other co-morbidities (79). Therefore, encouraging the inclusion of sufficient quantities of green leafy foods in a balanced diet aligns with the genetic background of most individuals while preserving the traditional intake of these foods (80) and minimizing the risk of chronic disease.

**Table 1:** Gene polymorphisms and their evolutionary mismatch among the admixed Mexican population.

Gene/allele	Lineage of allele predominance	Mesoamerican food culture	Evolutionary mismatch/disease
**High prevalence of MTHFR 677 T risk allele and TAS2R38/AVV bitter non-tasters**			
MTHFR/T (rs1801133)	Amerindian	High consumption of folate-rich bitter dark leafy greens	Low consumption of folate-rich foods
TAS2R38/AVV	Amerindian	Null consumption of distilled alcoholic beverages	High consumption of alcohol
**Differential prevalence of risk alleles for dyslipidemias**			
CD36/A (rs1761667)	Amerindian	A plant-based diet, high in complex carbohydrates, rich in MUFA and PUFA, and low in animal fat. Staple foods: maize, beans, chili, squash, tomato, chia, pumpkin seeds, amaranth, prickly pears, and cacao. No animal protein from cattle, pork, goat, or sheep.	High consumption of fatty-processed food
APOE/e2	European		High risk of hypertriglyceridemia and type II diabetes
APOE/e4	Amerindian		High risk of hypercholesterolemia
ABCA1/C (rs9282541)	Amerindian		High risk of hypoalphalipoproteinemia
**Carbohydrate metabolism**			
LCT/T-Lactase non-persistence (rs4988235)	Amerindian	No dairy products	High risk of hypertriglyceridemia
AMYA1 copy number	Amerindian/European	High consumption of complex carbohydrates: maize and beans	High consumption of simple sugars
**High prevalence of Taq A1 risk allele and addictive behaviors**			
DRD2/ANKK1/A1 (rs1800497)	Amerindian	Endemic food is high in complex carbohydrates, low in fats and sodium	Hepatopathogenic diet containing high calories, simple sugars, saturated fats, and sodium

MTHFR, Methylenetetrahydrofolate reductase; TAS2R38, Taste 2 Receptor Member 38; CD36, cluster of differentiation 36; APOE, Apolipoprotein E; ABCA1, Member 1 of the ATP-binding cassette transporter; LCT, lactase; DRD2/ANKK1, dopamine receptor D2/ ankyrin repeat and kinase-domain containing 1.

Another interesting example is the highly prevalent *TAS2R38* AVV haplotype that encodes the non-taster phenotype (81). This genetic trait aligns with the ability to tolerate bitter taste tolerances and the abundance of endemic bitter leafy vegetables mentioned before. Conversely, this haplotype is a risk factor for a higher consumption of alcohol among the Mexican population in modern times, which culturally has revolutionized over the centuries.

Similarly, when studying lipid-transporter genes, it is important to consider the differential allele distribution, within the admixed population (Table 1) (64). For example, the *ApoE* e2 and e4 alleles (65, 69), have been associated with hyperglyceridemia and hypercholesteremia, respectively. Thus, consuming chia, pumpkin seeds, and amaranth can offer a plant-based supply of mono-unsaturated fatty acids (MUFA) and poly-unsaturated fatty acids (PUFA) while inhibiting anti-inflammatory cell-signaling pathways that are frequently triggered in various chronic diseases (58). In line with these features, a recent study assessed the impact of specific nutrigenetic recommendations for 11 genetic variants associated with dyslipidemias, reducing blood lipids and low-grade inflammation in adults with excess weight (82).

Regarding carbohydrate metabolism, most Mexicans have inherited the lactose-intolerant trait, which is consistent with dairy animals not being part of the Mesoamerican ambient until after Spanish colonialization. Likewise, regulating the intake of complex carbohydrates from maize-based foods and legumes supports glucose homeostasis through enhanced insulin sensitivity based on the population’s average number of six *AMY1* copies. Also, the starch-resistant properties of these foods contribute to maintaining a healthy gut microbiota (64). Finally, the high prevalence of the DRD2/ANKK1 A1 allele has been associated with unhealthy food choices among the population (83). In conjunction, this information can help tailor nutrigenetic recommendations to address the prevalent dyslipidemias among Mexicans based on their unique genetic profiles.

Recently, the Genomex diet, containing most of the food staples of Mexico, was tested during a 6-month intervention nutrigenetic study, normalizing the anthropometric and biochemical profile of the study group (84) and improving auto-efficacy to maintain adherence to the nutritional plan (85). One significant effect was the 50% reduction of the HOMA-IR value after 3 months, which can be attributed to the components of the above dietary pattern. These results suggest that adherence to the Genomex diet decreases the risk of nutrition-related chronic diseases based on the millenary genetic adaptations that the Mexicans have inherited. Finally, an important feature of the Genomex diet is that the population culturally recognizes the recipes used to support this dietary program. Thus, nutritional recommendations based on the genetic profile of the population can be endorsed by “mom’s advice” by asking family members about the recipes of the food dishes comprising the staple indigenous foods.

In the same line of thought, studies carried out in East Asian populations where wild rice or other millet were consumed before their cultivation have revealed biological adaptations against the detrimental side effects of consuming high amounts of polished rise compared to other Asian populations (86). Thus, maintaining legendary dietary patterns may be the answer to preventing the risk of chronic diseases while conserving the local culinary culture.

Developing the genome-based nutrition strategy in Mexico has been challenging. The road to incorporating the principles of the Genomex diet into the Mexican clinical practice guidelines has faced skepticism from medical and nutrition associations. Another challenge is the food industry’s marketing consistently advocating for high-calorie ultra-processed foods and external dietary alternatives that impede food sovereignty and are not aligned with the Mexican population’s genetic makeup and food traditions. A marked rise in the consumption of ultra-processed foods in Mexico has been reported in the last three decades. Concurrently, there has been a decrease in the acquisition of unprocessed or minimally processed foods and processed culinary ingredients (87). Reversing this situation will require training and education in Genomic Nutrition to adequately prepare frontline clinicians, nutritionists, and other medical specialists to fight against these external factors and promote better eating patterns (88–92). In addition, research in complementary fields, including population genetics, food anthropology, social sciences, and evolutionary medicine studies, is crucial to provide an integrated framework defining the biological and cultural basis of a personalized or regionalized nutrition approach.

## 7 Conclusion

Will there be a time for the era of one-diet-fits-all to come to an end? We believe that the principles of the Genomex diet can aid in recovering and eating the traditional Mexican dietary ingredients while providing the nutrients that keep our adaptive genes healthy. Our mission is to educate health professionals and the general population on how to keep themselves nutritionally healthy, given our genetic legacy, regional food biodiversity, and food culture compatible with the Mexican population. Nonetheless, extrapolating the results throughout Mexico will require adjustments due to intraregional genetic and cultural differences, as mentioned before. In addition, implementing this approach in other Latin American countries will also require analyzing the regional prevalence of chronic diseases, prevailing risk alleles/traits, food culture, and other lifestyle factors (93–97) despite sharing a similar historical background of colonialism and globalization as Mexico. In this “weakness” lies the strength of eluding the one-diet-fits-all scheme and avoiding foreign alternatives while promoting entrepreneurism toward national eco-friendly agricultural and food industries aiming to contribute toward the world’s Sustainable Development Goals (98). Further research, training, teaching, and advocacy activities are required to advance toward a compelling personalized nutrition approach in Mexico to reclaim the benefits of a healthy diet.

## Data availability statement

The original contributions presented in the study are included in the article/supplementary material, further inquiries can be directed to the corresponding author.

## Author contributions

SR: Conceptualization, Investigation, Visualization, Writing – original draft, Writing – review & editing. LC-M: Investigation, Writing – review & editing, Visualization. LL-M: Investigation, Writing – review & editing, Visualization.
